# Effect of weight loss following Roux-en-Y gastric bypass on cancer risk: A Mendelian randomization study

**DOI:** 10.1097/MD.0000000000041351

**Published:** 2025-01-31

**Authors:** Jiaming Xue, Shuai Chen, Yu Wang, Yuwen Jiao, Dongmei Wang, Jie Zhao, Yan Zhou, Liming Tang

**Affiliations:** a Department of Graduate School, Dalian Medical University, Dalian City, Liaoning Province, China; b Department of Gastrointestinal Surgery, Affiliated Changzhou No. 2 People’s Hospital of Nanjing Medical University, The Third Affiliated Hospital of Nanjing Medical University, Changzhou Medical Center, Nanjing Medical University, Changzhou, People’s Republic of China.

**Keywords:** bariatric metabolic surgery, cancers, Mendelian randomization, Roux-en-Y gastric bypass

## Abstract

Cancer incidence and development are strongly correlated with obesity, however there is insufficient data to support a causal relationship between intentional weight loss and the prevention or promotion of cancer. We investigated the causal relationship between weight loss following Roux-en-Y gastric bypass (RYGB) and the incidence of 18 cancers using Mendelian randomization (MR). A genome-wide association studies (GWAS) data related to weight loss following RYGB from the GWAS catalog database were used as exposure, and GWAS data related to 18 cancers from the Medical Research Council integrative epidemiology unit open GWAS project were used as outcomes. In order to investigate the causal relationship between exposure and results, we used a two-sample MR approach. The primary analysis technique was inverse variance weighting, with weighted median, and MR-Egger regression utilized as supplemental techniques to confirm the findings. Heterogeneity and horizontal pleiotropy were investigated using a variety of sensitivity studies, including the Cochran *Q* test, MR-Egger regression pleiotropy test, MR pleiotropy residual sum and outlier, and leave-one-out analysis. We included a total of 4 single-nucleotide polymorphisms as instrumental variables through rigorous quality control screening. Under the limitations of Bonferroni correction threshold (*P* < 2.78 × 10^−3^), our results suggest that the weight loss following RYGB has a significant causal relationship with a reduced risk of breast (odds ratio [OR]: 0.784; 95% confidence interval [CI]: 0.762–0.808; *P* = 2.167e-58) and lung cancer (OR: 0.992; 95% CI: 0.987–0.997; *P* = .0023), and a potential causal relationship with a decreased risk of hematological cancer (OR: 0.9998462; 95% CI: 0.9997088–0.9999836; *P* = .028) and an increased risk of cervical cancer (OR: 1.000123; 95% CI: 1.0000313–1.000215; *P* = .009). Sensitivity analysis confirms the robustness of our analysis results. Genetically predicted weight loss following RYGB has significant causal effects in reducing the risk of breast and lung cancer. It also has potential benefits in lowering the risk of hemotological cancers and increasing the risk of cervical cancer. Considering the limitations of our study, the reliability of its results and the underlying mechanisms require further investigation.

## 1. Introduction

Over recent decades, obesity, defined as a body mass index (BMI) of 30 kg/m² or higher, has emerged as an escalating public health concern on a global scale. By 2016, the prevalence of obesity had reached over 670 million adults and 120 million children worldwide. Furthermore, more than 1.5 billion individuals were classified as overweight, with a BMI ranging from 25 kg/m² to 30 kg/m².^[1]^ As weight grows, type 2 diabetes, insulin resistance, hypertension, coronary heart disease, gallstones, stroke, fatty liver disease, sleep apnea syndrome, arthritis, and cancer are all considerably more common in overweight or obese people.^[2,3]^ At present, the main therapies used to manage obesity include bariatric surgery, medication, and lifestyle changes.^[4]^ Monotherapy with pharmacological agents often proves inadequate or presents limitations for individuals with severe obesity or associated comorbidities. In contrast, bariatric surgery demonstrates distinct benefits in terms of both therapeutic outcomes and recovery.^[4,5]^ Numerous studies have demonstrated that bariatric surgery represents the most rapid, sustained, and effective treatment for severe obesity, and it has been identified as one of the most effective treatments for type 2 diabetes.^[6,7]^ The 2 most popular procedures for bariatric surgery at the moment are sleeve gastrectomy (SG) and Roux-en-Y gastric bypass (RYGB).^[8]^ When evaluating surgical outcomes alone, RYGB is superior to SG in weight loss and improvement of complications, and is more effective in long-term weight loss.^[9,10]^

Meanwhile, the clinical, social, and economic cost of cancer on countries throughout the world has been steadily increasing in recent years. According to the World Health Organization’s Global Cancer Observatory (GLOBOCAN) reports that there would be 19.29 million new cancer cases worldwide in 2020, with a mortality rate of roughly 10.65%.^[11]^ According to survey data by Paul et al, approximately 40% of cancers in high-income countries are attributed to modifiable risk factors, meaning that these cancers are preventable. The most obvious modifiable cancer risk factors are tobacco and obesity, which cause more than half of all controlled cancers.^[12]^

Obesity and cancer have a close and complex association, including the following common pathways: (1) hyperinsulinemia, insulin resistance, and insulin-like growth factor-I signaling abnormalities; (2) chronic low-level inflammation and oxidative stress stimulation; (3) dysregulation of circulating sex hormone levels; (4) ectopic fat accumulation; (5) microenvironmental effects/cell perturbations; (6) pathophysiological alterations in adipokines/cytokines; (7) alterations in gut bacteria; and so on.^[13,14]^ Previous data from International Agency for Research on Cancer showed that obesity can increase the risk of 13 cancers, such as endometrial cancer, esophageal cancer, renal cancer, pancreatic cancer, hepatocellular carcinoma, cardiac cancer, meningioma, multiple myeloma, colorectal cancer, postmenopausal breast cancer, ovarian cancer, gallbladder cancer, and thyroid cancer.^[15,16]^ The evidence regarding the impact of intentional weight loss can lower this risk. According to some observational studies, bariatric surgery may significantly decrease the incidence rates of certain cancers, such as ovarian, colorectal, pancreatic, gallbladder, female breast cancers, and hepatocellular carcinoma. However, there appears to be minimal to no reduction in the risk of postoperative esophageal, thyroid, renal, and prostate cancers, among others.^[17–19]^ Conversely, research conducted by Mackenzie indicates an association between RYGB and an increased risk of colorectal cancer.^[20]^ Recent studies have observed a significant increase in the incidence of gastric and esophageal cancer following bariatric surgery in recent years. This trend may be attributable to the substantial rise in the number of bariatric procedures performed.^[21,22]^ According to the study by Ali et al, the reduction in the incidence of obesity-related cancers may be more closely associated with weight loss following RYGB rather than specific postoperative anatomical changes.^[23]^ Therefore, our hypothesis posits that weight loss resulting from RYGB surgery may influence the risk of certain cancers, suggesting a potential causal relationship between the 2.

Previous research has established that genetic factors significantly impact the weight loss outcomes following RYGB surgery, accounting for over 70% of the variability in weight loss outcomes.^[24]^ Consequently, genetics provides a valuable framework for exploring the casual relationship between intentional weight loss and a decreased risk of cancer development. Unlike certain observational studies, Mendelian randomization (MR) is more appropriately designed to ascertain causal effects. The MR approach is often conceptualized as “nature’s randomized controlled trial” due to the random assignment of genetic variations to offspring during gamete formation, in accordance with Mendel second law of independent assortment. As a result, MR effectively mitigates the influence of confounding variables and reverse causation. Compared to traditional analytical methods, MR offers enhanced capabilities for drawing robust causal inferences.

To our knowledge, no MR study has investigated the causal relationship between weight loss following RYGB and the reduced risk of cancer development. In this study, we designated the weight loss post-RYGB as the exposure variable and cancer incidence as the outcome variable. We employed a two-sample MR approach to examine the causal effect between these variables, aiming to enhance our understanding of the physiological changes induced by bariatric surgery and to contribute novel insights into cancer treatment, particularly for obesity-related malignancies.

## 2. Method

This study was conducted in accordance with the STROBE-MR checklist.^[25]^ The authors affirm that the research did not involve any animal or human subjects, utilizing solely publicly available de-identified data. Consequently, an ethical review was deemed unnecessary for this project.

### 2.1. MR design and data source

**Figure 1.**
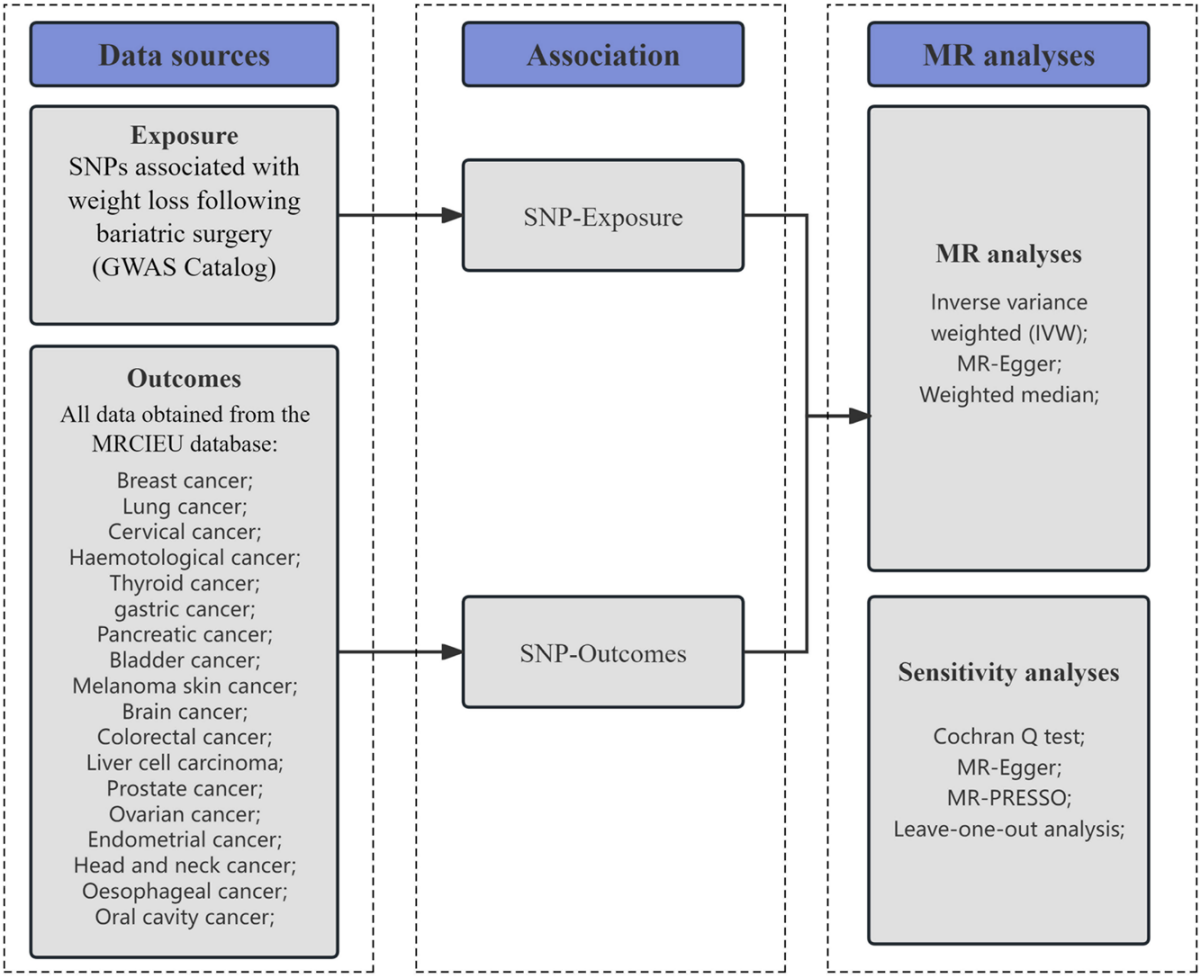
Design drawing of the study. SNP = single-nucleotide polymorphism; MR = Mendelian randomization; MR-PRESSO = MR pleiotropy residual sum and outlier.

We employed two-sample MR to investigate the relationship between weight loss following RYGB and the incidence of various cancers. Initially, we defined genetic variants (single-nucleotide polymorphisms, SNPs) linked to weight loss following RYGB as instrumental variables (IVs). Subsequently, we designated weight loss following RYGB as the exposure variable and multiple cancer types as the outcome variables. Comprehensive summary-level data encompassing all relevant SNPs were sourced from published genome-wide association studies (GWAS) or associated databases. Finally, we conducted a two-sample MR analysis utilizing 3 distinct MR methodologies: MR-Egger regression (MER), weighted median (WM), and inverse variance weighted (IVW) analysis. To ensure the robustness of the MR findings, we undertook a series of sensitivity analyses. Figure 1 illustrates the overall study design.

We identified a gene wide association with weight loss following gastric bypass in the GWAS catalog database (https://www.ebi.ac.uk/gwas/), which was uploaded by Hatoum et al and reported genetic variants associated with weight loss after surgical intervention.^[26]^ This study, ethically reviewed by Massachusetts General Hospital, involved 1020 patients who underwent gastric bypass surgery at Massachusetts General Hospital from February 2000 to October 2009. All patients satisfied the inclusion criteria and provided informed consent. The outcomes related to the GWAS were specified as the “percentage of weight loss at the weight nadir occurring at least 10 months post-surgery.”

For the GWAS outcome dataset in this study, the relevant genetic data of 18 cancers were obtained from several other studies. We extracted GWAS summary-level data for 18 cancers from Medical Research Council Integrative Epidemiology Unit Open GWAS project (https://gwas.mrcieu.ac.uk/). To mitigate potential bias arising from ethnic variability, we ensured that all study populations were of European ancestry. Furthermore, to the best of our knowledge, there was no overlap in samples between the GWASs pertaining to exposure and those concerning outcomes.

### 2.2. Selection of IVs

**Figure 2.**
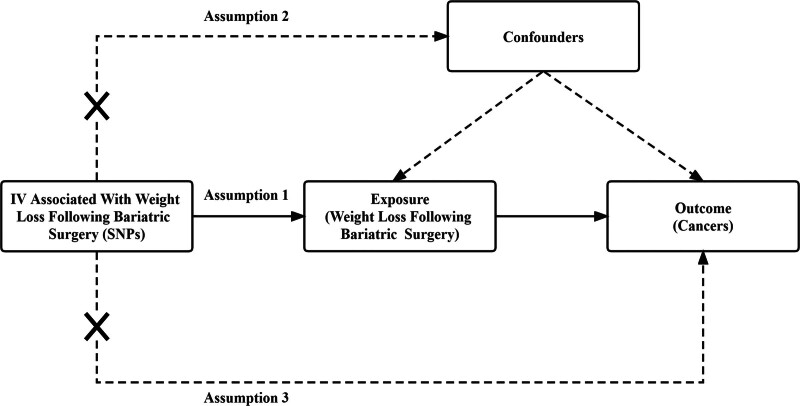
Core assumptions of Mendelian randomization. SNP = single-nucleotide polymorphism; IV = instrumental variables.

The classical MR model was used by us to evaluate the causality of exposure and outcome, which required the participation of effective IVs. As shown in Figure 2, in principle, the selection of IVs in the MR analyses process must meet 3 assumptions: (1) IVs must be related to exposure factors; (2) IVs must be independent of any other confounding factors; (3) IVs are associated with outcome factors only via exposure factors.^[27]^ In order to include an appropriate number of SNPs for subsequent analysis, based on recent relevant Mendelian research articles, we chose *P* < 1 × 10^−5^ as the threshold for significance.^[28,29]^ Then, we eliminated linkage disequilibrium by setting *r*^2^ > 0.001 within a 10,000 kb window. To mitigate the influence of weak IVs, we computed the *R*² and F-statistics, with the pertinent calculation formulas provided in Table S1, Supplemental Digital Content, http://links.lww.com/MD/O313.^[28,30–32]^ The SNPs with F < 10 were considered as weak IVs, and we removed them.^[33]^ Finally, we further matched these SNPs in phenotype-wide association studies (PheWAS, https://gwas.mrcieu.ac.uk/phewas/) to exclude SNPs directly associated with confounding factors or outcome factors.

### 2.3. MR analyses

To calculate the strength of the association between weight loss following RYGB and cancer, the IVW method was used as our primary analysis method. Based on the premise that all SNPS were free of horizontal pleiotrope, IVW can provide the most accurate estimate of causal effect by combining the Wald ratio of each SNP.^[34]^ When the results of Cochran Q statistic indicate the absence of heterogeneity (*P* ≥ .05), we used a fixed effects model, otherwise we used a random effects model. Furthermore, WM and MER were used as supplementary methods to verify the results. Even when up to 50% of SNPs are not valid, WM provides an unbiased estimate.^[35]^ MER is capable of assessing causal effects through its intercept and is also utilized to detect horizontal pleiotropy and heterogeneity.^[36]^ The odds ratio (OR) was utilized to quantify causal effects. A causal effect between the exposure and outcomes was inferred if the estimation directions of the 3 methods were consistent and the IVW method was statistically significant (*P* < .05).^[37,38]^

### 2.4. Sensitivity analyses

We employed a variety of methods for sensitivity analysis, including Cochran *Q* test, MER pleiotropy test, MR pleiotropy residual sum and outlier (MR-PRESSO), and leave-one-out analysis. Cochran *Q* statistic and corresponding *P*-values were used to assess the heterogeneity of each SNP.^[39]^ An MR-Egger intercept close to zero with a *P*-value ≥ .05 suggested the absence of horizontal pleiotropy.^[36]^ MR-PRESSO was employed to reassess pleiotropy and identify causal effects potentially influenced by outliers.^[38,40]^ A leave-one-out analysis was conducted to ascertain the impact of individual SNPs on the results by sequentially excluding each SNP.^[41]^ In instances where significant outlier values were identified through these analyses, they were removed, and the data were reanalyzed. The results were visualized using scatter plots, forest plots, and funnel plots.

### 2.5. Statistical analysis

All analyses were performed using the “TwoSampleMR” R package (v0.5.7), “MR-PRESSO” R package (v1.0) and the “MRInstruments” R package (v0.3.2) in the R software (v4.2.2). The production of the overall forest plot was completed through the “forestploter” R package (v1.1.1). In order to reduce the probability of false positive results, the Bonferroni correction threshold of the *P*-value < 2.78 × 10^−3^ (0.05/18) was preset to account for multiple testing. We regarded the *P*-value < 2.78 × 10^−3^ as strong evidence of a causal relationship between exposure and outcomes, while the *P*-value between 2.78 × 10^−3^ and .05 was suggestive evidence of the causal relationship. *P* < .05 was considered statistically significant.

## 3. Results

### 3.1. Participant characteristics and IVs

**Figure 3.**
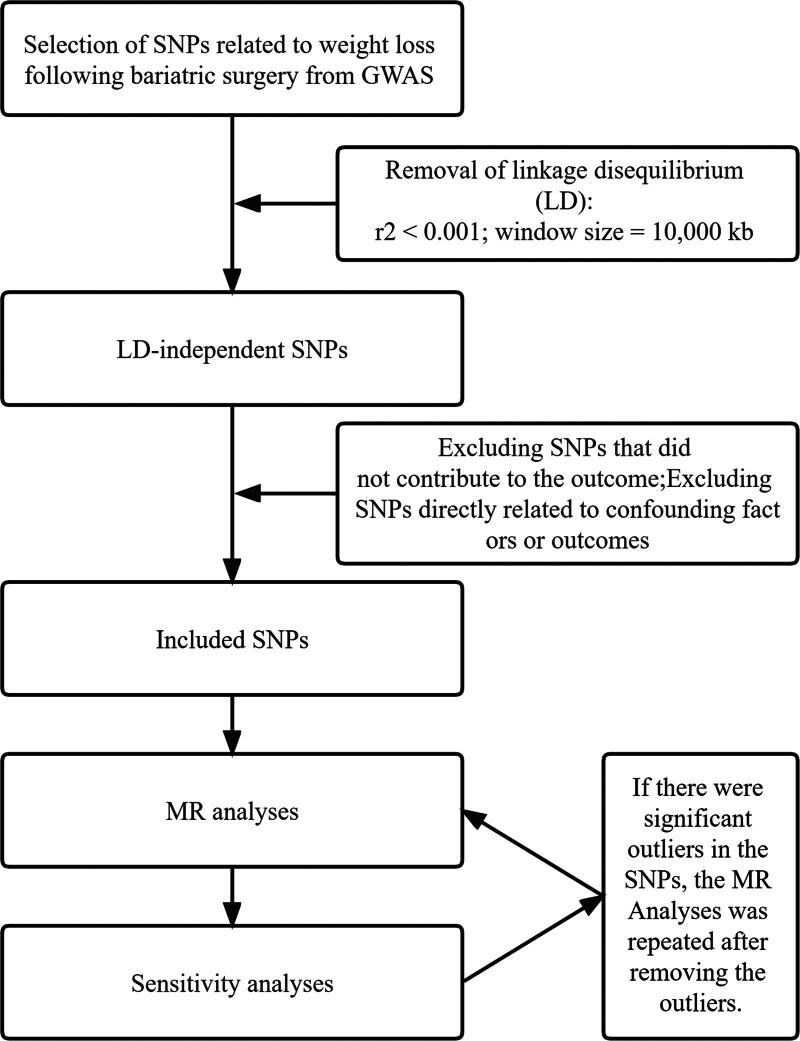
Flow chart of SNP screening. GWAS = genome-wide association studies; MR = Mendelian randomization; SNP = single-nucleotide polymorphism.

We selected 1 exposure dataset and 18 outcome datasets for our analysis. Comprehensive details regarding the data, including GWAS ID, sex, consortium, number of cases and controls, sample size, year, and population, are presented in Table 1. The process for screening relevant IVs is illustrated in Figure 3. Initially, 5 SNPs were identified as being associated with the exposure, all of which were retained following linkage disequilibrium pruning. However, upon cross-referencing with the PheWAS database (Table S2, Supplemental Digital Content, http://links.lww.com/MD/O313), we discovered that rs7129556 was directly associated with colon/sigmoid cancer and female genital organ cancer, leading to its exclusion from the cohort. The characteristic information of all SNPs can be found in Table 2. The F-statistic of each SNP was >10, and no weak IVs were found. The total F-statistic was 25.8.

**Table 1:** Detailed data information for all included datasets.

Variables	GWAS ID	Sex	Consortium	Cases	Controls	Sample size	Year	Population
Exposure								
Weight loss (gastric bypass surgery)	NA	M & F	NA	1020	NA	1020	2013	European
Outcomes								
Breast cancer	ebi-a-GCST007236	NA	NA	46,785	42,892	89,677	2015	European
Lung cancer	ieu-a-985	M & F	TRICL	23,848	16,605	40,453	NA	European
Cervical cancer	ieu-b-4876	Females	NA	563	198,523	199,086	2021	European
Hemotological cancer	ieu-b-4878	M & F	UK Biobank	4552	372,016	376,568	2021	European
Thyroid cancer	finn-b-C3_THYROID_GLAND	M & F	FinnGen consortium	989	217,803	NA	2021	European
Gastric cancer	finn-b-C3_STOMACH	M & F	FinnGen consortium	633	218,159	NA	2021	European
Pancreatic cancer	finn-b-C3_PANCREAS	M & F	FinnGen consortium	605	218,187	NA	2021	European
Bladder cancer	ieu-b-4874	M & F	UK Biobank	1279	372,016	373,295	2021	European
Melanoma skin cancer	ieu-b-4969	M & F	UK Biobank	3751	372,016	375,767	2021	European
Brain cancer	ieu-b-4875	M & F	UK Biobank	606	372,016	372,622	2021	European
Colorectal cancer	ieu-b-4965	M & F	UK Biobank	5657	372,016	377,673	2021	European
Liver & bile duct cancer	ieu-b-4915	M & F	UK Biobank	3350	372,016	372,366	2021	European
Prostate cancer	ukb-d-C3_PROSTATE	M & F	UK Biobank	6321	354,873	361,194	2018	European
Ovarian cancer	ieu-b-4963	Females	UK Biobank	1218	198,523	199,741	2021	European
Endometrial cancer	ebi-a-GCST006464	Females	NA	12,906	108,979	121,885	2018	European
Head and neck cancer	ieu-b-4912	M & F	UK Biobank	1106	372,016	373,122	2021	European
Oesophageal cancer	ieu-b-4960	M & F	UK Biobank	740	372,016	372,756	2021	European
Oral cavity cancer	ieu-b-4961	M & F	UK Biobank	357	372,016	372,373	2021	European

F = females, M = males, TRICL = transdisciplinary research in cancer of the lung.

**Table 2:** Characteristics of SNPs related to weight loss following weight loss surgery.

SNP	Chr	Pos	Gene	EA	OA	EAF	F-statistics	Beta	SE	*P*-value
rs10515808	5	159820931	C1QTNF2	A	C	0.1143	25.3	-3.08	0.6122449	4e-07
rs17702901	15	92930411	ST8SIA2	A	G	0.0278	29.1	-6.64	1.2295918	7e-08
rs7158359	14	89594295	FOXN3	G	C	0.1988	20.6	-2.27	0.5000000	5e-06
rs7185923	16	51245487	SALL1	C	T	0.5030	21.4	-1.89	0.4081633	4e-06

Chr = chromosome, EA = effect allele, EAF = effect allele frequency, OA = other allele, Pos = position, SE = standard error, SNP = single-nucleotide polymorphism.

### 3.2. MR analyses

In our study, we employed 3 methodologies, IVW, WM, and ME, to investigate the causal relationships between exposure and outcomes. The initial MR analysis, as detailed in Table S3, Supplemental Digital Content, http://links.lww.com/MD/O313, reveals a significant association between weight loss following RYGB and the incidence of breast, lung, cervical, and hematological cancers. However, subsequent application of the MR-PRESSO test identified horizontal pleiotropy among SNPs in the breast cancer cohort. Consequently, SNPs rs17702901 and rs7185923 were excluded based on MR-PRESSO feedback. The results of the heterogeneity test indicated significant heterogeneity in the breast cancer (Q = 10.34, *P* = .0013) and prostate cancer (Q = 8.742, *P* = .032) (Table S4, Supplemental Digital Content, http://links.lww.com/MD/O313). Consequently, the 2 datasets were analyzed using the IVW random effects model, whereas the remaining datasets were analyzed using the fixed effects model. The additional outcomes did not exhibit significant heterogeneity or horizontal pleiotropy. Table 3 and Figure 4 present the final results of the MR analysis. It can be observed that the IVW estimates of breast cancer OR = 0.784, 95% confidence interval (CI) = 0.762–0.808, *P*-value = 2.167e-58), lung cancer (OR = 0.992, 95% CI = 0.987–0.997, *P*-value = .0023), cervical cancer (OR = 1.000123, 95% CI = 1.0000313–1.000215, *P*-value = .009) and hemotological cancer (OR = 0.9998462, 95% CI = 0.9997088–0.9999836, *P*-value = .028) are significant (*P* < .05), and the directions of IVW, WM, and MER estimates are consistent. Breast cancer and lung cancer are conserved after the Bonferroni correction level (2.78 × 10^−3^) is applied. Therefore, the findings from the MR analysis offer robust evidence indicating that weight loss following RYGB surgery is associated with a reduced risk of breast and lung cancers. Additionally, the analysis provides suggestive evidence that such weight loss may decrease the risk of hematological cancers while potentially increasing the risk of cervical cancer. The results of the sensitivity analysis are detailed in Table 4. Notably, the MER did not reveal any significant intercept. The results of leave-one-out analysis (Figures S1–S18, Supplemental Digital Content, http://links.lww.com/MD/O312), scatter plots (Figures S19–S36, Supplemental Digital Content, http://links.lww.com/MD/O312), forest plots (Figures S37–S54, Supplemental Digital Content, http://links.lww.com/MD/O312), and funnel plots (Figures S55–S72, Supplemental Digital Content, http://links.lww.com/MD/O312) are presented in additional materials.

**Table 3:** The results of MR analyses.

Outcomes	N	IVW		WM		ME	
		OR (95% CI)	*P*	OR (95% CI)	*P*	OR (95% CI)	*P*
Breast cancer	2	0.784 (0.762–0.808)	2.167e-58	NA	NA	NA	NA
Lung cancer	4	0.992 (0.987–0.997)	.0023	0.990 (0.984–0.997)	.003	0.984 (0.972–0.997)	.051
Cervical cancer	3	1.000123 (1.0000313–1.000215)	.009	1.000116 (0.9999857–1.000246)	.081	1.000223 (0.9998791–1.000566)	.425
Hemotological cancer	3	0.9998462 (0.9997088–0.9999836)	.028	0.9998886 (0.9997090–1.0000683)	.224	0.9998269 (0.9994254–1.0002285)	.553
Thyroid cancer	4	0.994 (0.972–1.0116)	.591	0.984 (0.957–1.012)	.268	0.970 (0.899–1.047)	.515
Gastric cancer	4	0.995 (0.968–1.022)	.705	0.988 (0.956–1.021)	.463	0.964 (0.910–1.022)	.346
Pancreatic cancer	4	0.978 (0.951–1.006)	.117	0.980 (0.950–1.011)	.202	0.980 (0.922–1.041)	.580
Bladder cancer	3	0.9999767 (0.9999030–1.000051)	.537	0.9999707 (0.9998740–1.000068)	.553	1.0000146 (0.9998103–1.000219)	.9111558
Melanoma skin cancer	3	1.0000154 (0.9998836–1.000147)	.809	1.0000562 (0.9998992–1.000213)	.483	0.9999984 (0.9996001–1.000397)	.995
Brain cancer	3	0.9999941 (0.9999432–1.000045)	.821	0.9999992 (0.9999383–1.000060)	.979	0.9999561 (0.9998468–1.000065)	.576
Colorectal cancer	3	1.000146 (0.9999938–1.000299)	.060	1.000186 (1.0000045–1.000367)	.045	1.000126 (0.9996771–1.000575)	.680
Liver & bile duct cancer	3	1.000018 (0.9999795–1.000057)	.356	1.000023 (0.9999780–1.000069)	.314	1.000025 (0.9999414–1.000108)	.666
Prostate cancer	4	1.000068 (0.9998150–1.000321)	.599	1.000118 (0.9999155–1.000321)	.253	1.000495 (1.0001438–1.000847)	.110
Ovarian cancer	3	1.0000302 (0.9998955–1.000165)	.661	0.9999897 (0.9998068–1.000173)	.912	0.9997121 (0.9994227–1.000002)	.302
Endometrial cancer	4	1.000329 (0.9925985–1.008120)	.934	1.002520 (0.9924106–1.012733)	.626	1.006137 (0.9878408–1.024771)	.581
Head and neck cancer	3	1.0000274 (0.9999587–1.000096)	.435	1.0000383 (0.9999576–1.000119)	.352	0.9999484 (0.9998010–1.000096)	.617
Oesophageal cancer	3	1.000052 (0.9999959–1.000108)	.069	1.000053 (0.9999896–1.000116)	.102	1.000027 (0.9999066–1.000148)	.734
Oral cavity cancer	3	1.0000078 (0.9999687–1.000047)	.694	1.0000013 (0.9999550–1.000048)	.956	0.9999721 (0.9998881–1.000056)	.633

CI = confidence interval, IVW = inverse variance weighting, MER = MR-Egger regression, WM = weighted median, MR = Mendelian randomization, OR = odds ratio.

**Table 4:** The results of sensitivity analyses.

Outcomes	Heterogeneity		MR-Egger pleiotropy test		MR-PRESSO	
	Q	*P*	Intercept	*P*	RSSobs	*P*
Breast cancer	10.34	.0013	NA	NA	NA	NA
Lung cancer	2.597	.458	0.022	.466	4.26	.554
Cervical cancer	4.244	.120	-0.0003	.638	NA	NA
Hemotological cancer	1.875	.392	6.43e-5	.932	NA	NA
Thyroid cancer	6.330	.097	0.081	.552	10.389	.216
Gastric cancer	1.728	.631	0.104	.358	3.655	.607
Pancreatic cancer	0.116	.990	-0.007	.947	0.217	.991
Bladder cancer	1.946	.378	-0.00012	.752	NA	NA
Melanoma skin cancer	2.218	.330	5.662e-05	.940	NA	NA
Brain cancer	0.997	.608	0.00013	.583	NA	NA
Colorectal cancer	1.896	.388	6.90e-05	.935	NA	NA
Liver & bile duct cancer	0.372	.830	-2.12e-05	.893	NA	NA
Prostate cancer	8.742	.032	-0.001	.119	16.679	.101
Ovarian cancer	5.941	.051	0.001	.248	NA	NA
Endometrial cancer	2.349	.503	-0.018	.566	3.795	.556
Head and neck cancer	1.631	.442	0.0002	.446	NA	NA
Oesophageal cancer	0.214	.898	8.27e-05	.728	NA	NA
Oral cavity cancer	0.919	.632	0.0001	.519	NA	NA

MR-PRESSO = MR pleiotropy residual sum and outlier.

**Figure 4.**
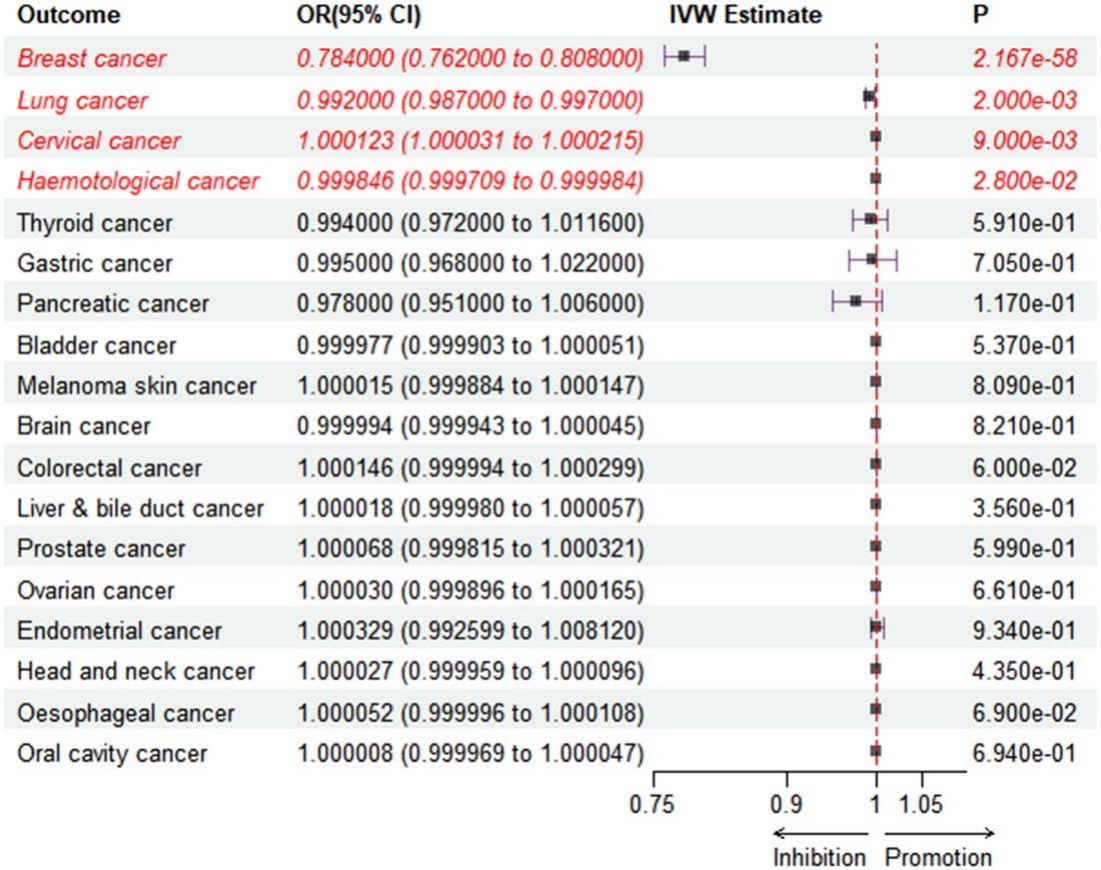
Forest plot of the causal relationship between exposure and outcome derived from IVW analysis. CI = confidence interval; IVW = inverse variance weighting; OR = odds ratio.

## 4. Discussion

We employed two-sample MR to assess the causal relationship between weight loss following RYGB and the risk of different cancers based on the summary-level data from GWAS. Our findings indicate a significant causal association between genetically predicted weight loss post-RYGB and a reduced risk of breast and lung cancers. Additionally, the analysis provides suggestive evidence that such weight loss may decrease the risk of hematological cancers while potentially increasing the risk of cervical cancer.

In light of the established association between weight gain and increased cancer risk, researchers have initiated investigations into the potential of intentional weight loss to mitigate this effect. Prior observational and cohort studies examining nonsurgical weight loss strategies have predominantly concentrated on enhancing exercise regimens and dietary management.^[42]^ Luo et al demonstrated that intentional weight loss can substantially decrease the risk of obesity-related cancer risk (11 types of cancer, including ovarian, liver, endometrial, breast, pancreatic, etc), whereas unintentional weight fluctuations do not exhibit a similar impact.^[43]^ In addition, the risk reduction of endometrial cancer^[44,45]^ and breast cancer^[46–48]^ has also been confirmed by many other studies. Nonetheless, when investigating the relationship between conservative weight loss and cancer risk, several challenges must be addressed: (1) conservative weight loss has the characteristics of a long period of time, and the weight loss effect is not obvious and difficult to maintain, which weakens the association between weight loss and cancer risk; (2) conservative weight loss is difficult to distinguish between intentional weight loss and unintentional weight loss^[49]^; (3) increased physical activity during conservative weight loss might independently reduce cancer risk^[50]^; (4) dietary modifications could also independently lower cancer risk.^[51,52]^ Considering these limitations, it appears more compelling to investigate the association between weight loss and cancer risk by employing bariatric surgery as a method for weight reduction.

At the moment, bariatric surgery has become an important clinical model to explore the relationship between intentional weight loss and subsequent cancer risk.^[53]^ There are many operative techniques for bariatric surgery, with RYGB being the second most common procedure, and its weight-loss effect has been widely recognized.^[54]^ Although RYGB results in more substantial anatomical alterations compared to SG, Ali et al proposed that the reduction in cancer risk is primarily attributable to the weight loss itself, rather than the physiological changes associated with these anatomical modifications.^[23]^ Numerous cohort and observational studies have explored the association between RYGB and cancer risk, although their findings are debatable. Khalid and Clapp reported that individuals who underwent RYGB experienced a reduction in overall cancer risk compared to those who did not undergo bariatric surgery.^[55,56]^ Mackenzie suggested that RYGB significantly reduced the risk of hormone related cancers (breast cancer, endometrial cancer, and prostate cancer) after surgery, but increased the risk of colorectal cancer.^[20]^ But Khalid findings that RYGB can reduce an individual’s risk of developing colorectal cancer refute his claim.^[55]^ Notably, Hussan research suggests that RYGB reduces the risk of colorectal cancer in women compared to the control group, while it significantly elevates the risk in men.^[57]^ Meanwhile, Khalid et al also found that RYGB can significantly reduce the risk of lung cancer,^[55]^ which is consistent with our MR analysis results.

Our findings corroborate existing evidence indicating that weight loss following RYGB surgery is associated with a decreased risk of breast, lung, and hematologic cancers, while potentially increasing the risk of cervical cancer. These results align, to some extent, with prior research. In terms of cancer prevention, RYGB may be achieved through mechanisms such as reducing excessive inflammation, improving insulin resistance, and regulating levels of sex hormones and adipokines.^[23,58–60]^ Our findings suggest that weight loss following RYGB may have a limited causal impact on the heightened risk of cervical cancer. Despite an extensive search, we were unable to find any studies or reports indicating an increased prevalence of cervical cancer post-bariatric surgery. However, we discovered that Avgerinos et al article said that obesity may be a protective factor for cervical cancer.^[13]^ In a study on cervical cancer patients undergoing concurrent radiotherapy and chemotherapy, the authors observed significant improvements in local control and overall survival outcomes in obese patients. And this difference cannot be explained by the use of diabetes or metformin. They demonstrated that differences in the PI3K/AKT pathway are responsible for the different responses of obese and nonobese cervical cancer patients to standard radiotherapy and chemotherapy.^[61]^ Meanwhile, Jungles et al also stated that cervical cancer patients with greater BMI were more sensitive to standard chemoradiotherapy, which could be explained by fat cells releasing more monounsaturated and diunsaturated free fatty acids and activating beta oxidation.^[62,63]^ But obviously, it needs additional confirmation in the future.

## 5. Limitations

Despite our efforts to control for confounding variables throughout the study, it is important to acknowledge the inherent limitations of our conclusions. Firstly, even though we selected the largest dataset for the exposure dataset, the number of SNPs included was still minimal. To adequately expand the number of SNPS, we selected a somewhat looser criteria, which aligns with generally accepted practice. The assessment of all SNPs found no weak IVs, and a series of sensitivity studies found no obvious abnormalities, suggesting that the selected genetic variants were sufficiently robust. Although the MR-Egger and MR-PRESSO tests did not indicate the presence of horizontal pleiotropy, the potential for such pleiotropy cannot be entirely excluded. Consequently, future research with a larger sample size will be necessary to more accurately evaluate the impact of genetic factors on weight loss following bariatric surgery. Secondly, despite our efforts to exclude unsuitable SNPs following the screening of the PheWAS database, we were unable to rule out all potential confounders due to the incompleteness of the data in the published dataset. Furthermore, the sex ratio of our exposure cohort differs significantly from that of the partial outcome cohorts. However, due to the limitations of the raw data, we were unable to conduct a deeper examination, which may introduce partial bias. Finally, to mitigate the impact of racial disparities, we selected populations of European ancestry for both the exposure and outcome cohorts. This choice may limit the generalizability of the results to other populations. Furthermore, even within the same ancestry group, variations in geography and socioeconomic background among the cohort populations may influence the interpretation of the results. Consequently, although MR has been demonstrated to be an effective method for assessing the causal relationship between weight loss following RYGB and cancer, its findings require validation through additional research based on experimental studies.

## 6. Conclusion

In summary, we explored the relationship between weight loss following RYGB and 18 cancers using genetic data and two-sample MR methods. Our results demonstrate that weight loss following RYGB reduces the risk of breast and lung cancer and suggest that it may play a potential role in reducing the risk of hemotological cancer and increasing the risk of cervical cancer.

## Acknowledgments

The authors thank Home for Researchers editorial team (https://www.home-for-researchers.com/) for polishing the article. The authors also thank all investigators for sharing these data.

## Author contributions

**Conceptualization:** Jiaming Xue, Dongmei Wang, Liming Tang.

**Data curation:** Jiaming Xue, Shuai Chen.

**Formal analysis:** Jiaming Xue, Yu Wang.

**Investigation:** Yu Wang, Yan Zhou.

**Methodology:** Jiaming Xue, Shuai Chen, Jie Zhao, Liming Tang.

**Project administration:** Yan Zhou.

**Resources:** Dongmei Wang, Yan Zhou, Liming Tang.

**Software:** Shuai Chen, Yu Wang.

**Supervision:** Yu Wang, Yuwen Jiao, Jie Zhao.

**Validation:** Jiaming Xue, Yuwen Jiao, Jie Zhao.

**Writing – original draft:** Jiaming Xue, Shuai Chen.

**Writing – review & editing:** Dongmei Wang, Jie Zhao, Liming Tang.
